# Hybridization alters red deer gut microbiome and metabolites

**DOI:** 10.3389/fmicb.2024.1387957

**Published:** 2024-05-03

**Authors:** Limin Wei, Bo Zeng, Bo Li, Wei Guo, Zhenqiang Mu, Yunong Gan, Yanhong Li

**Affiliations:** ^1^Chongqing Key Laboratory of High Active Traditional Chinese Drug Delivery System, Chongqing Medical and Pharmaceutical College, Chongqing, China; ^2^College of Pharmacy, Chongqing Medical University, Chongqing, China; ^3^Farm Animal Genetic Resources Exploration and Innovation Key Laboratory of Sichuan Province, Sichuan Agricultural University, Chengdu, China; ^4^College of Resources and Environment, Aba Teachers University, Aba, China; ^5^School of Laboratory Medicine, Chengdu Medical College, Chengdu, China; ^6^Key Laboratory of Endemic and Ethnic Diseases, Ministry of Education & Key Laboratory of Medical Molecular Biology of Guizhou Province, & Collaborative Innovation Center for Prevention and Control of Endemic and Ethnic Regional Diseases Co-constructed by the Province and Ministry, Guizhou Medical University, Guiyang, Guizhou, China

**Keywords:** red deer, hybridization, gut microbiome, pilose antler, multi-omics technologies

## Abstract

The host genes play a crucial role in shaping the composition and structure of the gut microbiome. Red deer is listed as an endangered species by the International Union for the Conservation of Nature, and its pilose antlers have good medicinal value. Hybridization can lead to heterosis, resulting in increased pilose antler production and growth performance in hybrid deer. However, the role of the gut microbiome in hybrid deer remains largely unknown. In this study, alpha and beta diversity analysis showed that hybridization altered the composition and structure of the gut microbiome of the offspring, with the composition and structure of the hybrid offspring being more similar to those of the paternal parents. Interestingly, the LefSe differential analysis showed that there were some significantly enriched gut microbiome in the paternal parents (such as *g_Prevotellaceae UCG-003*, *f_Bacteroidales RF16 group; Ambiguous_taxa*, etc.) and the maternal parents (including *g_Alistipes*, *g_Anaerosporobacter*, etc.), which remained significantly enriched in the hybrid offspring. Additionally, the hybrid offspring exhibited a significant advantage over the parental strains, particularly in taxa that can produce short-chain fatty acids, such as *g_Prevotellaceae UCG-003*, *g_Roseburia, g_Succinivibrio*, and *g_Lachnospiraceae UCG-006*. Similar to bacterial transmission, metagenomic analysis showed that some signaling pathways related to pilose antler growth (“Wnt signaling pathway,” “PI3K Akt signaling pathway,” “MAPK signaling pathway”) were also enriched in hybrid red deer after hybridization. Furthermore, metabolomic analysis revealed that compared with the paternal and maternal parents, the hybrid offspring exhibited significant enrichment in metabolites related to “Steroid hormone biosynthesis,” “Tryptophan metabolism,” “Valine, leucine and isoleucine metabolism,” and “Vitamin B metabolism.” Notably, the metagenomic analysis also showed that these metabolic pathways were significantly enriched in hybrid deer. Finally, a correlation analysis between the gut microbiome and metabolites revealed a significant positive correlation between the enriched taxa in hybrid deer, including the *Bacteroidales RF16 group*, *Prevotellaceae*, and *Succinivibrio*, and metabolites, such as 7α-hydroxytestosterone, L-kynurenine, indole, L-isoleucine, and riboflavin. The study contributes valuable data toward understanding the role of the gut microbiome from red deer in hybridization and provides reference data for further screening potential probiotics and performing microbial-assisted breeding that promotes the growth of red deer pilose antlers and bodies, development, and immunity.

## 1 Introduction

The gut microbiome plays a crucial role in regulating the metabolic energy, the immune system, and host activation of the host, as well as maintaining the intestinal physiological functions, which are essential for overall host health (Gill et al., 2006; Magnus et al., 2013; Deng et al., 2023). Previous research showed the potential regulatory mechanisms of the production of proteins in milk at the rumen microbial and host levels through metagenomics and metabolomic analyses of the bovine rumen, as well as serum metabolomics (Xue et al., 2020). Another study revealed that members of *Selenomonas* and *Succinivibrionaceae* mutually promoted each other in dairy cows with high feed efficiency, thereby providing important ecological functions and active carbohydrate metabolism. Random forest machine learning was used to identify six rumen metabolites derived from carbohydrates as potential metabolic markers. These markers could differentiate between highly efficient and poorly efficient microbial communities with an accuracy of 95.06% (Xue et al., 2022). Kokou et al. (2018) conducted a study on the gut microbiome of cold tolerant tropical tilapia through subculture breeding and found that the gut microbiome in the cold tolerant tilapia also exhibited “cold tolerance” characteristics similar to those of the host, with higher resilience to temperature changes. This suggests that the gut microbiome are shaped by selection processes similar to those of the host. Therefore, the microbial community may contribute to shaping host production parameters. A deeper understanding of this process may enable us to adjust gut microbiome of animal hosts, thereby enhancing their productivity (Bergamaschi et al., 2020).

Red deer (*Cervus elaphus*) are singular economic animals that are endangered by the International Union for the Conservation of Nature. The pilose antlers are a valuable traditional Chinese medicine. Studies have shown that pilose antlers have anti-inflammatory, anti-fatigue, antioxidant and anti-osteoporosis effects and can enhance immunity (Zha et al., 2013; Ma et al., 2017; Pham et al., 2022; Pei et al., 2023). Therefore, the primary purpose of raising deer is to produce pilose antlers. However, pilose antlers are expensive due to the long breeding cycle and high costs of feed. The cross-breeding of red deer not only increases pilose antler production and economic benefits but also improves the growth, reproduction, and disease resistance of their offspring (Yang and Qin, 2013). The genes of the host are closely related to the composition and structure of the gut microbiome (Turpin et al., 2016). The gut microbiome co-evolves with the host and can be stably transmitted to subsequent generations with the host (Bjork et al., 2011; Langergraber et al., 2012). Fan et al. (2020) reported that the host genes can alter the structure of the gut microbiome through interactions, thereby further affecting the blood glucose levels, weight, and non-esterified fatty acid content in the calves. Previous studies have shown that the primary mode of the transmission of gut microbiome is vertical transmission (Bjork et al., 2011; Langergraber et al., 2012; Moeller et al., 2018), and hybridization plays an important role in shaping the gastrointestinal microbiome of the offspring (Kreisinger et al., 2014; Wang et al., 2015).

Several previous studies have demonstrated that the gut microbiome of red deer play a crucial role in energy metabolism, environmental adaptation, and health (Qian et al., 2017; Glendinning et al., 2021; Yan et al., 2022). However, the effects of hybridization on the gut microbes and metabolites of red deer offspring, as well as the potential role of the gut microbiome in the growth of host pilose antlers, remain unclear. This study conducted a comprehensive analysis that utilized multiomics approaches to compare the changes in the gut microbiome and metabolites of deer before and after hybridization. The aim of this study was to further identify microbial and metabolite features associated with the production of pilose antlers, providing reference data for understanding the potential role of the gut microbiome in enhancing deer pilose antler yield.

## 2 Materials and methods

### 2.1 Ethics statement

All animal experiments were approved by the Institutional Animal Care and Use Committee of the Chongqing Medical and Pharmaceutical College, Chongqing, China (KCB-2220031).

### 2.2 Experimental objects and sample collection

**Figure 1:**
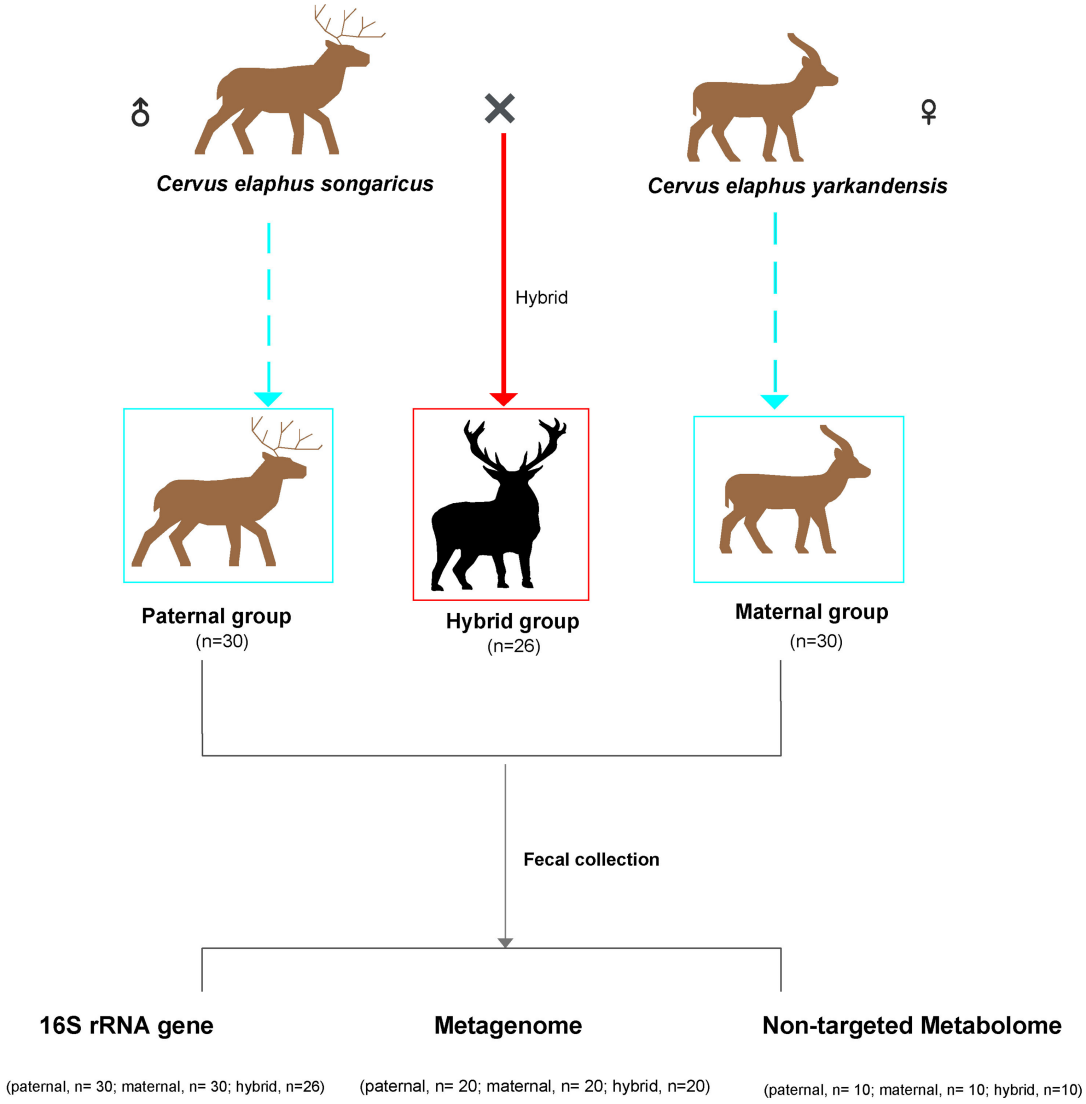
Schematic overview of the experimental design. Sample correspondence information is shown in Supplementary Table S1.

Fecal samples of *Cervus elaphus songaricus* (paternal, *n* = 30) and *Cervus elaphus yarkandensis* (maternal, *n* = 30), as well as fecal samples from hybrid red deer (hybrid, *n* = 26), were collected from a red deer farm in Urumqi, Xinjiang, China (Figure 1). This experiment used *Cervus elaphus songaricus* as the paternal parent and *Cervus elaphus yarkandensis* as the maternal parent to naturally mate. The hybrid deer inherits 50% of the paternal lineage from *Cervus elaphus songaricus* and 50% of the maternal lineage from *Cervus elaphus yarkandensis*. Supplementary Table S1 showed the specific information of the samples, including gender, age, and corresponding detection methods. The animals were anesthetized using Deer Sleeping Ling (xylazine hydrochloride injection) to facilitate the quick collection of fresh fecal samples from the anus. Subsequently, the deer were quickly injected with Deer Wake Ling (*Nikethamide*) to revive them. Deer Sleeping Ling and Deer Wake Ling are produced by Qingdao Hanhe Animal and Plant Pharmaceutical Co., Ltd. in China. Fresh fecal samples were placed in sterile centrifuge tubes and quickly placed in liquid nitrogen for storage. All the animals participating in the experiment were healthy, with no history of antibiotics use. In addition, the feeding environment and composition of the diets of all the animals were consistent. The specific information on these diets is shown in Supplementary Table S2.

**Figure 2:**
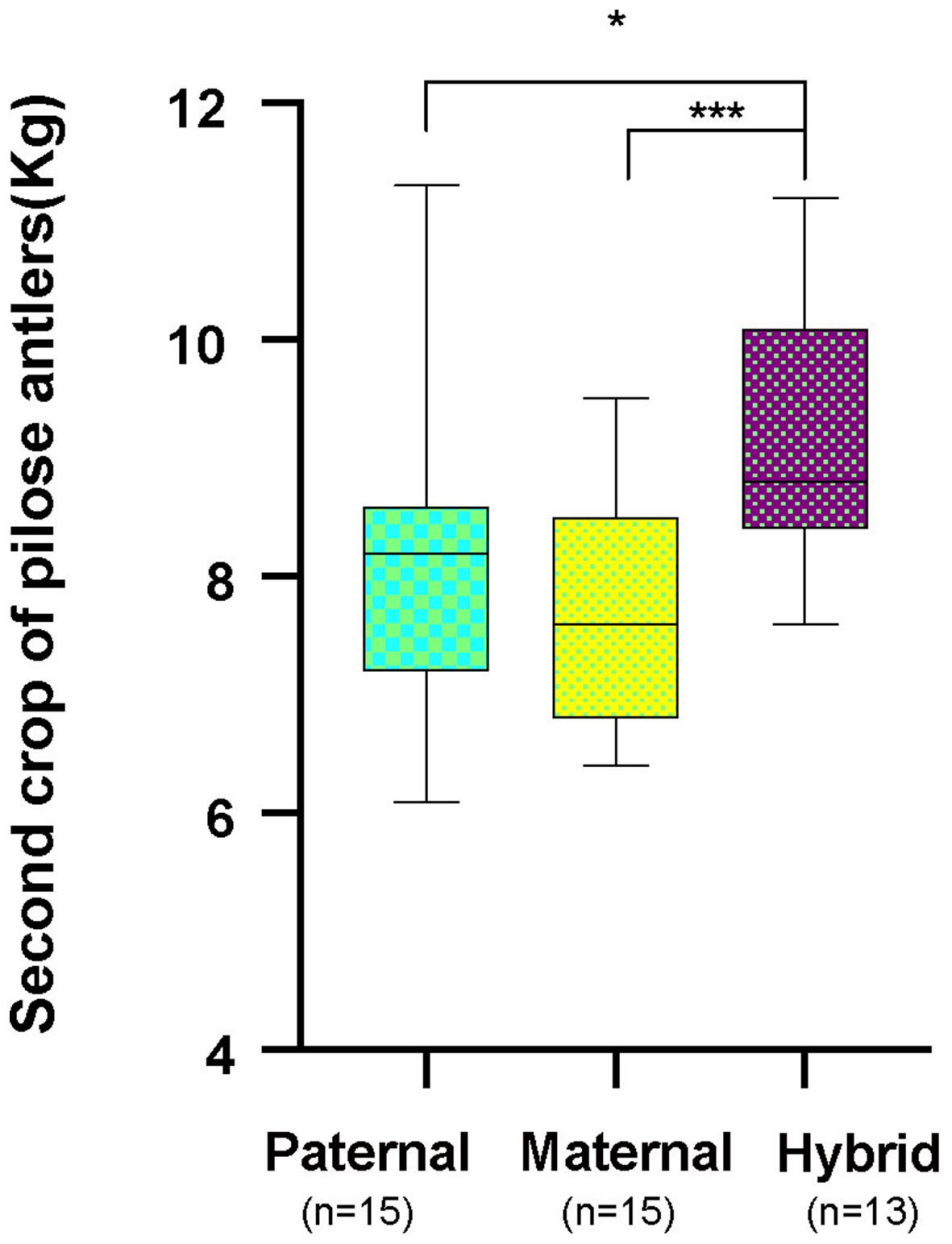
Annual production of second crop of pilose antlers by the parent red deer and their hybrid. Paternal group: *n* = 15; maternal group, *n* = 15; hybrid group, *n* = 13. *p < 0.05, ***p < 0.001.

*Cervus elaphus songaricus* can tolerate cold better and has a higher rate of feed collection, while *Cervus elaphus yarkandensis* tolerates heat better, has a rough feeding tolerance, and is resistant to diseases. These hybrid offspring exhibit the dominant traits of both parents, which include an increase in the production of pilose antlers, improved fecundity, enhanced disease resistance, and strong adaptability to the environment. Red deer pilose antler is harvested twice a year. The first harvest is called the “first crop of pilose antlers” and the second harvest is called the “second crop of pilose antlers.” This study collected data on the weights of second crop of pilose antlers from 15 male *Cervus elaphus songaricus* deer (paternal group), 15 male *Cervus elaphus yarkandensis* deer (maternal group), and 13 male hybrid red deer (hybrid group) in the experiment. The average production of pilose antlers by the parents ranged from 7.82 to 8.01 kg, while that of the hybrid offspring was 9.04 kg. The weight of deer pilose antlers produced by hybrid offspring was significantly greater than that of the parents (Figure 2; *p* < 0.05, Student T test).

### 2.3 Extraction of the total DNA from the microbial genomes and bacterial 16S rRNA gene V3-V4 region sequencing

QIAamp Power Fecal DNA Kit (Qiagen, Germantown, United States) was used to extract the total DNA of the microbial genome from the fecal samples. The DNA concentration was determined using a NanoDrop spectrophotometer (Thermo Scientific) and further quantified via agarose gel electrophoresis. The qualified DNA samples were then sent to Novogene Bioinformatics Technology, Co., Ltd. (Beijing, China), which used the high-throughput HiSeq 2,500 platform of Illumina (San Diego, CA, United States) to construct the library and sequence the V3-V4 region of the 16S rRNA genes of the microorganisms. Briefly, the V3-V4 region of the 16S rRNA gene was amplified using the 341F/806R barcoded primer pair (341F, CCT AYG GGR BGC ASC AG, 806R: GGA CTA CNN GGG TAT CTA AT).During PCR amplification, FastStart high fidelity (Roche, Basel, Switzerland) and Phusion enzyme mixes were used in a Phusion^®^ High-Fidelity PCR Master Mix (New England Biolabs, Ipswich, MA, United States). The PCR steps included predenaturation at 98°C for 30 s, followed by 30 cycles of 94°C for 10 s, 50°C for 20 s, 72°C for 20s, and a final extension at 72°C for 90 s. Negative controls were incorporated during DNA extraction and amplification to mitigate any potential contamination from the swabs and kits. After PCR amplification and gel electrophoresis, the PCR products were purified using the Qiagen Gel Extraction Kit (Qiagen, Germany). Subsequently, sequencing libraries were generated using the TruSeq^®^ DNA PCR-Free Sample Preparation Kit (Illumina, United States).

### 2.4 Analysis of the bacterial 16S rRNA data

The open software Quantitative Insights Into Microbial Ecology 2 (QIIME2, v. 2023.9; Bolyen et al., 2019) was used to process the 16S rRNA V3-V4 sequencing data. First, DADA2 (Callahan et al., 2016) was used to perform quality control processing on the original data, filtering out noise and error sequences. The removal of chimeric and redundant sequences was performed in this step, followed by the selection of operational taxonomic units (OTUs) based on a 97% similarity threshold specific to the 16S rRNA V3-V4 region. Next, the OTUs were taxonomically annotated using the Silva database (silva_138.1_release). Finally, the alpha diversity of the microbial community was evaluated based on calculations of the observed OTUs index. Moreover, Bray-Curtis distance algorithm was used to analyze the beta diversity of the microbial communities.

### 2.5 Metagenomic analysis

A total of 20 fecal samples from each of the three groups were randomly selected and sent to Novogene Bioinformatics Technology Co., Ltd., which used an Illumina NovaSeq 6,000 platform for metagenome sequencing following Qubit fluorimetry (Thermo Fisher Scientific, Waltham, MA, United States) test. The quality of data was ensured using Sickle software (v. 1.33) to control the raw data, and the reads that contained low-quality bases and N base numbers that reached 5% were removed. Subsequently, BWA v. 0.7.1 software was used to compare the red deer genome reference sequence, and the host genome sequence was removed (Li and Durbin, 2009). Megahit v. 1.2.2 software (Li D. et al., 2016) was used to splice the dehosted reads to generate contigs. The open reading frames (ORFs) of the spliced data sequences were predicted using Prodigal v. 2.6.1 software (Hyatt et al., 2010). The construction of a non-redundant reference gene set (Zou et al., 2019) was performed using CD-HIT software (Fu et al., 2012), which ensured that redundant information would not appear in the subsequent analyses. Biopython was used to translate the nucleotide sequences of the non-redundant gene sets into amino acid sequences. Finally, the amino acid sequences were uploaded to the official website of the Kyoto Encyclopedia of Genes and Genomes (KEGG) for functional annotation of this non-redundant gene set (Kanehisa et al., 2016).

### 2.6 Fecal untargeted metabolome (LC–MS) analysis

The metagenome sequencing backup samples were randomly selected, with 10 samples from each of the paternal, maternal, and hybrid groups sent to Novogene Bioinformatics Technology, Co., Ltd. for a non-targeted metabolomics analysis via liquid chromatography-mass spectrometry (LC–MS). The samples were pretreated and analyzed by chromatography and mass spectrometry using an Agilent 1,290 Infinity LC ultrahigh pressure liquid chromatograph (Agilent Technologies, Santa Clara, CA, United States) and a Triple TOF 6600+ mass spectrometer (AB Sciex, Framingham, MA, United States), respectively.

The experiment began by injecting the experimental samples and quality control (QC) samples into the column at a flow rate of 0.3 mL/min, while the temperature was adjusted to a constant 4°C. The eluent used in the chromatographic column consisted of A and B where A was composed of water, ammonium acetate, and ammonia, and B was acetonitrile. The solvent gradient was then established with the following specific steps: starting with 85% B, which was eluted within 1 min. The content of B was gradually reduced to 65% using a linear gradient within 0.1 min. In the next 3 min, the content of B was reduced to 40% by a linear gradient. In the subsequent 3 min, the content of B increased to 85% by a linear gradient. Finally, 85% B was maintained, followed by a 5 min equilibration step.

Finally, the chemicals isolated in these steps were analyzed by mass spectrometry to obtain detailed information about the non-targeted metabolites in the sample. This series of steps contributes to a comprehensively understanding the metabolic composition of the sample and provides reliable data for subsequent metabolomics research.

### 2.7 Processing of the LC–MS data

The mzCloud, mzVault and MassList databases were used to identify the matching peaks to obtain as much metabolite information as possible. The XCMS program was used to preprocess the raw data, including the alignment of peaks, correction of the retention time and extraction of the peak areas. Accurate molecular weights and molecular formulae of the compounds were then determined, and the metabolites were identified using these databases according to their fragment ions, collision energies, and other information. The coefficient of variance was set to <30% as the threshold, and the metabolites that were highly stable were screened for subsequent normalization and quantitative analyses. Finally, the KEGG database was used to annotate the final metabolites identified to understand their functional characteristics and distribution in the organism.

### 2.8 Statistical analysis

The significance of the differences in alpha-diversity and beta-diversity was analyzed using the Mann–Whitney test and analysis of similarity(ANOSIM), respectively. Linear discriminant analysis effect size (LEfSe; LDA value >2) was used to differentially analyze the differential bacterial flora, KEGG categories, and KEGG pathways between the groups. In the partial least squares-discriminant analysis (PLS-DA) model, the variable importance of projection (VIP) value of the first principal component was used to evaluate the rate of contribution of the metabolites, and the fold difference (FC) was used to identify the differential metabolites. Combined with the *p* value detected by the LefSe, the thresholds were set to VIP > 1.0, FC > 2.0 or FC < 0.5, and *p* < 0.05 was used to identify the differential metabolites. R software (3.6.0) was used to generate visual displays, draw box plots, conduct a principal coordinate analysis (PCoA), and create the PLS-DA, histograms, annotated distribution charts and other results. The R packages used included VennDiagram, ggplot2, and ggpubr. A Spearman correlation analysis was used to explore the correlations between significantly different metabolites and bacterial groups. The network diagram was drawn using Cytoscape (v.3.6.1). Using MicrobioAnalyst 2.0 (Lu et al., 2023) to analyze core microorganisms at the genus level.^1^ Set the thresholds as: sample prevalence (%) > 60, relative abundance (%) > 0.05.

## 3 Results

### 3.1 16S rRNA sequencing results

A 16S rRNA V3-V4 sequencing analysis of the fecal samples from the paternal, maternal, and hybrid groups resulted in the yielded 5,054,008 high-quality reads, with an average of 54,733 reads per sample. A total of 111,451 OTUs were identified (Supplementary Table S3).

**Figure 3:**
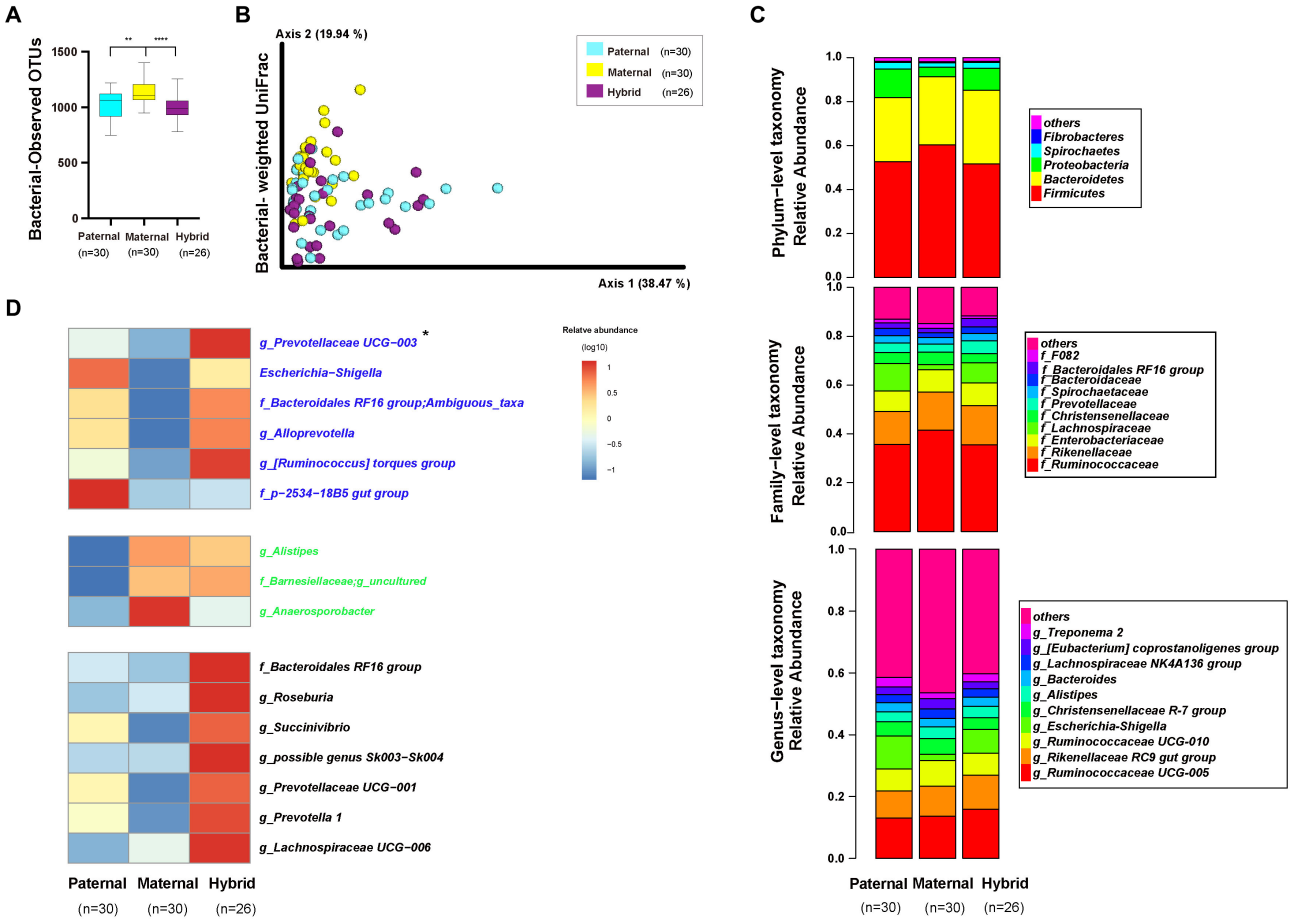
Diversity, composition, and differential analyses of the gut microbiome from the paternal, maternal and hybrid groups. Paternal group: *n* = 30; maternal group, *n* = 30; hybrid group, *n* = 26. **(A)** Observed OTUs index. Cyan, yellow, and purple bars represent paternal, maternal, and hybrid, respectively. ***p < 0.001, ****p < 0.0001, Mann–Whitney U-test. **(B)** Bacteria weighted UniFrac PCoA. Cyan, yellow, and purple circles represent the paternal, maternal, and hybrid bacterial communities, respectively. ANOSIM, *p* < 0.05. **(C)** Phylum-, family-, and genus-level distributions of the 16S rRNA sequences in the paternal, maternal, and hybrid groups (top 5 phylum-level classifications, top 10 family-level classifications, and top 10 genus-level classifications, respectively). **(D)** Heatmap of the genus-level differential bacterial communities between the paternal, maternal and hybrid groups. Blue font indicates that paternal and hybrid were more significant than those in the maternal group. Green font indicates that maternal and hybrid were more significant than the paternal group; Black font indicates that hybrid was more significant than both the paternal and maternal groups; Additionally, an asterisk (*) denotes that the significance of the hybrid group was higher than that of both the paternal and maternal groups.

An alpha-diversity analysis of the 16S rRNA V3-V4 sequencing results revealed a significantly higher observed OTUs index in the maternal group than in the paternal and hybrid groups (Figure 3A; Mann–Whitney test, *p* < 0.001; Supplementary Table S4). However, no significant difference was observed between the paternal and hybrid groups. Using the weighted UniFrac distance algorithm to calculate beta diversity, significant differences were found among all groups, except between the paternal and hybrid groups, where the difference was not significant (Figure 3B; ANOSIM, *p* < 0.05; Supplementary Table S4). This indicates that, compared with the gut microbiome from the maternal deer, the gut microbiome structure of the paternal-hybrid was more similar.

The top 5 phyla that were the most abundant in the paternal, maternal, and hybrid groups included *Firmicutes*, *Bacteroidetes*, *Proteobacteria*, *Spirochaetes*, and *Fibrobacteres*, collectively accounting for an average of 98.29% (Figure 3C). The maternal group exhibited a significantly higher relative abundance of *Firmicutes* than the paternal and hybrid groups (Figure S1A; Mann–Whitney test, *p* < 0.01), while the paternal and hybrid groups had a significantly higher relative abundance of *Proteobacteria* than the maternal group (Figure S1C). In hybrids, the relative abundance of *Bacteroidetes* was significantly higher than that in the paternal group, while the relative abundances of *Proteobacteria* and *Spirochaetes* were significantly higher than those in the maternal group (Figures S1B,D).

The top 10 genera at the family level in the paternal, maternal, and hybrid groups accounted for approximately 86.2% of the total abundance, with the top 3 genera being *Ruminococcaceae*, *Rikenellaceae*, and *Lachnospiraceae* (Figure 3C). Relative abundances of *Ruminococcaceae* and *F082* in the maternal groups were significantly higher than those in the paternal and hybrid groups (Figures S2A,B). Additionally, the relative abundance of *Rikenellaceae* in the maternal and hybrid groups were significantly higher than those in the paternal group (Figure S2C). The relative abundances of *Enterobacteriaceae* and *Prevotellaceae* in the paternal and hybrid groups were significantly higher than those in the maternal groups (Figures S2D,E). Moreover, the relative abundance of the *Bacteroidales RF16 group* in the hybrids was significantly higher than that in the paternal and maternal groups (Figure S2F), while the abundance of *Christensenellaceae* was significantly lower than in those in these groups (Figure S2G). *Lachnospiraceae*, *Bacteroidaceae*, and *Spirochaetaceae* showed no significant changes during the hybridization process (Figures S2H–J).

The top 10 OTUs at the genus level, including *Ruminococcaceae UCG-005*, *Rikenellaceae RC9 gut group*, *Ruminococcaceae UCG-010*, *Escherichia-Shigella*, *Christensenellaceae R-7 group*, *Alistipes*, *Bacteroide*s, *Lachnospiraceae NK4A136 group*, *[Eubacterium] coprostanoligenes group*, and *Treponema 2*, collectively represented approximately 57.28% of the total abundance (Figure 3C). Through the analysis of core microorganisms, it was found that the three groups of core microorganisms were consistent, namely *Ruminococcaceae UCG-005*, *Rikenellaceae RC9 gut group*, and *Ruminococcaceae UCG-010* (Figure S3).

The LefSe software was used to analyze the genera-level microbial communities (those with a relative abundance >0.1%) that exhibited differentially abundant among the paternal, maternal, and hybrid groups (Figure 3D). Some microbial communities regularly changed during the process of hybridization. Particularly, the abundances of *g_Prevotellaceae UCG-003*, *Escherichia-Shigella*, *f_Bacteroidales RF16 group; Ambiguous_taxa*, *g_Alloprevotella*, *g_[Ruminococcus] torques group*, *f_p-2534-18B5 gut group,* and *g_uncultured bacterium* were significantly higher in the paternal and hybrid groups compared with those in the maternal group. In contrast, the abundances of *g_Alistipes*, *f_Barnesiellaceae*; *g_uncultured*, and *g_Anaerosporobacter* were significantly higher in the maternal and hybrid groups than the paternal group. Interestingly, the hybrids had significantly higher abundances of some genera of gut microbiome than both the paternal and maternal groups in certain genera, including *g_Prevotellaceae UCG-003*, *f_Bacteroidales RF16 group;g_uncultured bacterium*, *g_Roseburia*, *g_Succinivibrio*, *g_possible genus Sk003-Sk004*, *g_Prevotellaceae UCG-001*, *g_Prevotella 1*, and *g_Lachnospiraceae UCG-006* (Figure 3D).

### 3.2 KEGG functional analysis

The Illumina NovaSeq 6,000 platform generated a total of 2,889,253,724 paired raw reads through sequencing. After removing low-quality and contaminating sequences, 2,884,673,142 clean reads were obtained. Subsequently, after splicing the high-quality sequences, 25,542,652 contigs were generated, and 41,803,416 ORFs were obtained (Supplementary Table S5).

The analysis of the top 20 KEGG categories revealed that “Global and overview maps,” “Carbohydrate metabolism,” and “Amino acid metabolism” ranked as the top 3, collectively constituting approximately 44.91% of the total content (Figure S4A). The relative abundances of “Global and overview maps,” “Metabolism of cofactors and vitamins,” “Metabolism of terpenoids and polyketides,” and “Digestive system” were significantly higher in the paternal and hybrid groups compared with the maternal group. Alternatively, “Carbohydrate metabolism,” “Metabolism of other amino acids,” “Glycan biosynthesis and metabolism,” “Signal transduction,” “Cell growth and death,” “Immune system,” and “Excretory system” had significantly higher relative abundances in the maternal and hybrid groups compared with the paternal group. Additionally, “Biosynthesis of other secondary metabolites,” “Lipid metabolism,” and “Transport and catabolism” were significantly more abundant in the hybrid group compared with both the maternal and paternal groups (Figure S4B).

**Figure 4:**
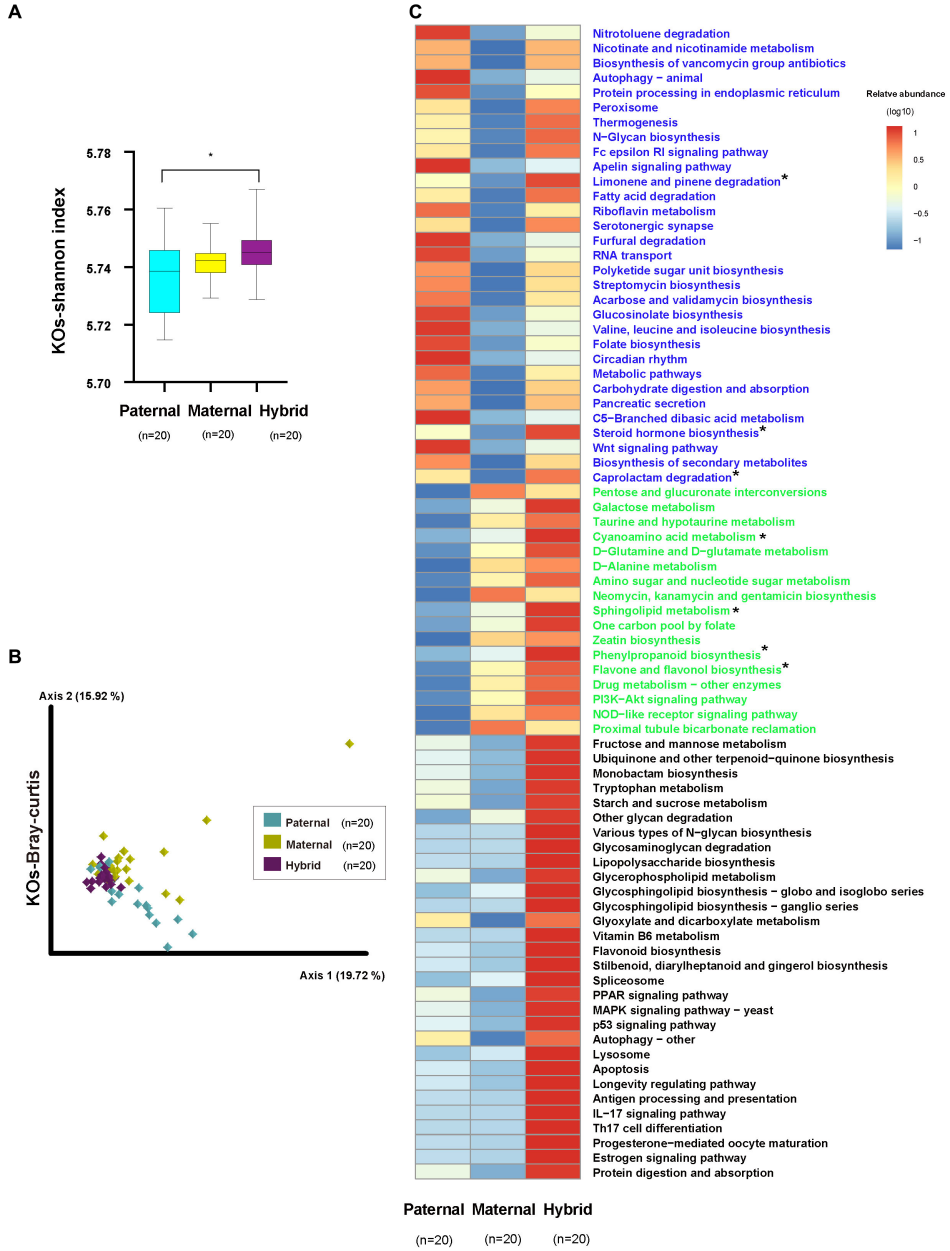
KEGG functional analysis. Paternal group: *n* = 20; maternal group, *n* = 20; hybrid group, *n* = 20. **(A)** Shannon index of the KOs. Cyan, yellow, and purple bars, paternal, maternal, and hybrid, respectively. *p < 0.05, Mann–Whitney U-test. **(B)** KOs of a Bray-Curtis PcoA. Cyan, yellow, and purple diamonds indicate paternal, maternal and hybrid KOs, respectively. **(C)** Heatmap of different KEGG pathways. Blue font indicates that paternal and hybrid were more significant than those in the maternal group. Green font indicates that maternal and hybrid were more significant than the paternal group; Black font indicates that hybrid was more significant than both the paternal and maternal groups; Additionally, an asterisk (*) denotes that the significance of the hybrid group was higher than that of both the paternal and maternal groups.

The analysis of the metagenomic sequencing results revealed a significantly higher Shannon index for the KEGG Orthologs (KOs) in the hybrid group compared with the paternal group (Whitney test, *p* < 0.01; Figure 4A; Supplementary Table S4). However, no significant difference was observed between the paternal and maternal groups. Additionally, the analysis of the Bray-Curtis distances for the KOs revealed significant differences among the three groups (ANOSIM, *p* < 0.01; Figure 4B; Supplementary Table S4).

The analysis of differential pathways also revealed systematic changes, consistent with the findings from the microbial community analysis (Figure 4C). A total of 30 pathways exhibited significantly higher levels in the hybrid and paternal groups compared with the maternal group, including the “Wnt signaling pathway,” “Nitrotoluene degradation,” “Nicotinate and nicotinamide metabolism,” and “Biosynthesis of vancomycin group antibiotics.” A total of 18 pathways showed significantly higher levels in the hybrid and maternal groups compared with the paternal group, including “PI3K-Akt signaling pathway,” “Pentose and glucuronate interconversions,” “Galactose metabolism,” “Taurine and hypotaurine metabolism,” “Cyanoamino acid metabolism,” and “D-Glutamine and D-glutamate metabolism.” Additionally, 30 pathways were significantly higher in the hybrid group than in both the paternal and maternal groups, including “MAPK signaling pathway – yeast,” “Fructose and mannose metabolism,” “Ubiquinone and other terpenoid-quinone biosynthesis,” “Monobactam biosynthesis,” “Tryptophan metabolism,” and “Starch and sucrose metabolism.”

### 3.3 Non-targeted fecal metabolome analysis

Untargeted fecal metabolome analysis revealed that the top 5 fecal metabolites of the paternal, maternal, and hybrid groups belonged to KEGG classifications including “Global and overview maps,” “Lipid metabolism,” “Amino acid metabolism,” “Digestive system,” and “Nucleotide metabolism” (Figure S5A). A PLS-DA revealed that significant separation of fecal metabolite groups of the paternal, maternal, and hybrid into three clusters ((Figure S5B); ANOSIM, *p* < 0.01).

**Figure 5:**
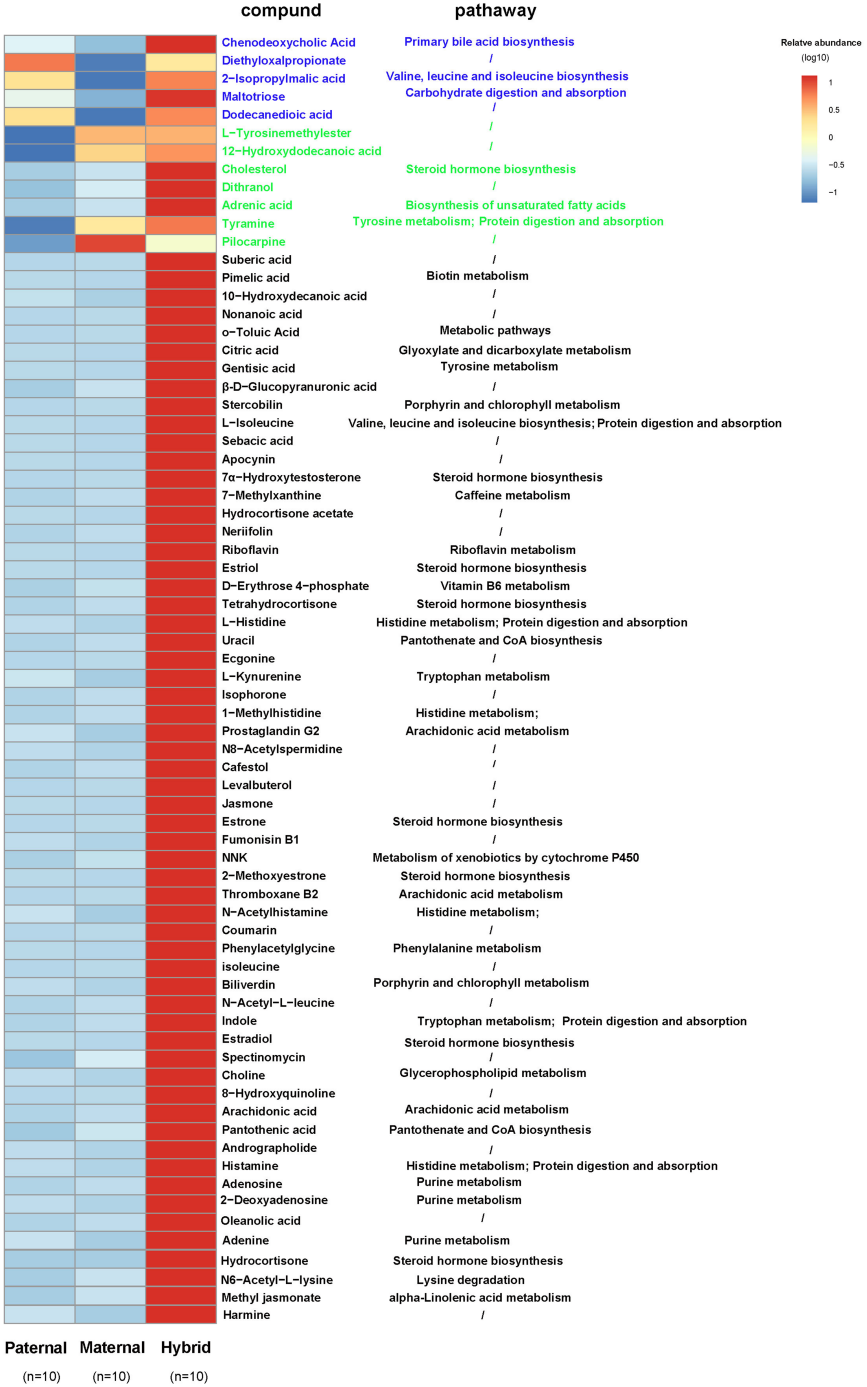
Heatmap of the differentially abundant metabolites. Paternal group: *n* = 10; maternal group, *n* = 10; hybrid group, *n* = 10.Blue font indicates that paternal and hybrid were more significant than those in the maternal group. Green font indicates that maternal and hybrid were more significant than the paternal group; Black font indicates that hybrid was more significant than both the paternal and maternal groups; Additionally, an asterisk (*) denotes that the significance of the hybrid group was higher than that of both the paternal and maternal groups.

A comparative analysis of the differences among the three groups of metabolites revealed that five metabolites in the hybrid group exhibited similar to those in the paternal group and significantly higher than those in the maternal group. For instance, chenodeoxycholic acid, 2-isopropylmalic acid, and altotriose were enriched in “Primary bile acid biosynthesis,” “Valine, leucine and isoleucine biosynthesis,” and “Carbohydrate digestion and absorption,” respectively (Figure 5). In addition, seven metabolites in the hybrid group shared similarities with those in the maternal group but were significantly higher than those in the paternal group, including cholesterol, adrenic acid, and tyramine, which were enriched in “Steroid hormone biosynthesis,” “Biosynthesis of unsaturated fatty acids,” and “Tyrosine metabolism,” respectively (Figure 5). Interestingly, 59 metabolites, including citric acid, L-isoleucine, riboflavin, 7α-hydroxytestosterone, D-erythrose 4-phosphate, L-kynurenine, and arachidonic acid, were significantly more abundant in the hybrid group than in the maternal and paternal groups. These metabolites were primarily enriched in metabolic pathways, such as “Glyoxylate and dicarboxylate metabolism,” “Valine, leucine and isoleucine degradation,” “Riboflavin metabolism,” “Steroid hormone biosynthesis,” “Vitamin B6 metabolism,” “Biotin metabolism,” “Pantothenate and CoA biosynthesis,” “Tryptophan metabolism,” and “Arachidonic acid metabolism.” (Figure 5).

### 3.4 Analysis of the association between bacteria and the metabolites

**Figure 6:**
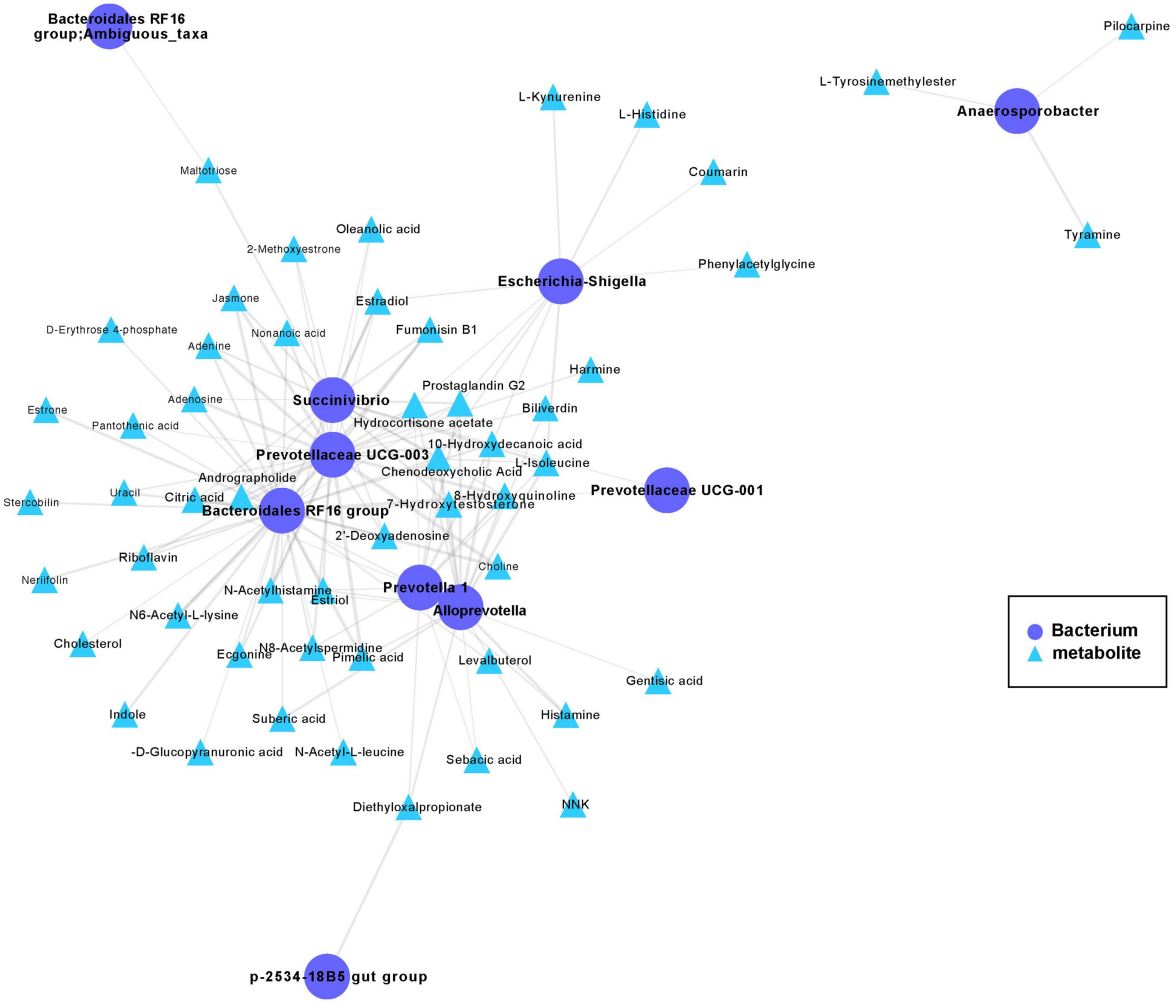
The co-occurrence networks of the metabolites and bacteria enriched in the hybrid. Select metabolites and bacteria with higher abundance in the hybrid group than in the paternal and maternal groups (Figures 3D, 5) for paired Spearman correlation analysis and network visualization (*r* > 0.8 and *p* < 0.01; paternal group: *n* = 10; maternal group: *n* = 10; hybrid group: *n* = 0; bacterial and metabolite samples correspond one-to-one.). Circles represent enriched bacterium, triangles represent enriched metabolites. Nodes are connected by pairwise interactions (links). The weight of the link indicated a strong Pearson correlation (*r* > 0.8, *p* < 0.01), which was shared between metabolites and genes. Readers are suggested to read the web version of the article for interpretation of the high-resolution figures.

To further study the mechanistic interactions between the gut microbiome and metabolites within the hybrid, the hybrid group exhibited a higher abundance of both metabolites and bacteria than paternal group and the maternal group (Figures 3D, 5) were selected for a pairwise Spearman correlation analysis and visualization of the network (*r* > 0.8 and *p* < 0.01; Figure 6). The network diagram can be primarily divided into two clusters. The *Bacteroidales RF16 group*, *Prevotellaceae UCG-003*, *Succinivibrio*, *Alloprevotella*, *Prevotella 1*, and *Escherichia-Shigella*, among others, were clustered in one group, while *Anaerosporobacter* was positioned in another cluster. Notably, *Bacteroidales RF16 group*, *Prevotellaceae UCG-003*, *Succinivibrio*, *Alloprevotella*, *Prevotella 1*, and *Escherichia-Shigella* closely grouped together, resulting in the highest positive correlations. Additionally, they were associated with the most correlated metabolites.

The abundance of the *Bacteroidales RF16 group* was significantly positively correlated with the metabolites, including pimelic acid, pantothenic acid, 7α-hydroxytestosterone, citric acid, riboflavin, D-erythrose 4-phosphate, prostaglandin G2, choline, N6-acetyl-L-lysine, and indole. Notably, the abundance of *Prevotellaceae UCG-003* showed a positive correlated with the metabolites, including pantothenic acid, citric acid, L-isoleucine, riboflavin, prostaglandin G2, N6-acetyl-L-lysine, choline, and estradiol among others. The abundance of *Succinivibrio* showed significant positive correlations with the metabolites, including citric acid, L-isoleucine, prostaglandin G2, estriol, chenodeoxycholic acid, and others in the metabolic pathways. Additionally, the abundance of *Alloprevotella* was primarily positively correlated with the metabolites, including pimelic acid, gentisic acid, prostaglandin G2, L-isoleucine, 7α-hydroxytestosterone, and estriol. The abundance of *Prevotella 1* significantly positively correlated with the metabolites, including chenodeoxycholic acid, citric acid, prostaglandin G2, L-isoleucine, and estriol.

## 4 Discussion

The gut microbiome co-evolve with the host and can be stably transmitted to subsequent generations with the host (Bjork et al., 2011; Langergraber et al., 2012). Studies on inbred mice have showed that the gut microbiome are primarily transmitted through vertical transmission. The gut microbiome of the offspring of inbred mice can be traced back to the ancestral generation of mice and can be continuously and stably transmitted to the 10th generation (Moeller et al., 2018). Furthermore, host genetics have been demonstrated to play an important role in the formation of hybrid gastrointestinal microbiome (Buhnik-Rosenblau et al., 2011; Kreisinger et al., 2014; Wang et al., 2015). Studies have shown that the rumen microbiome of the hybrid offspring differs from that of their parents, suggesting that the significant impact of host genetics on the rumen microbiome may stem from vertical transmission (Li Z. et al., 2016). Furthermore, through alpha diversity analysis, it was found that the Observed OTUs index of hybrid deer did not significantly exceed that of the parents. This is similar to the results of our previous study on the intestinal microbiota of hybrid wild boars over four generations, where there were no significant changes in the Shannon index and Observed OTUs during the hybridization process (Wei et al., 2023). Additionally, a study also found that hybrid Batun (Berkshire × Tunchang) pigs showed no significant changes in Simpson, Chao1, ACE, and Shannon indices of alpha diversity compared to purebred Tunchang pigs (He et al., 2023). Both studies observed changes in beta diversity after hybridization, consistent with our experimental results. These data suggest that hybridization may alter the gut microbial composition of offspring with less impact on taxa numbers. Furthermore, our study revealed that the gut microbiota structure of paternal-hybrid deer was more similar to that of the paternal group compared to the maternal group, possibly due to a greater influence of the paternal genome on the offspring gut microbiota (Fan et al., 2020).

Interestingly, evidence of gene recombination following hybridization was found (Mallet, 2007), and a similar recombination phenomenon appeared to occur in the gut microbiome of hybrid red deer. The hybrid offspring produced significantly more pilose antlers than the parents, and they also had more advantageous growth, reproductive performance, and immunity (Yang and Qin, 2013). Previous research from our laboratory on the gut microbiome of inbred and hybrid pigs revealed that the composition and function of gut microbiome of offspring also showed “decline” and “dominance” phenomena similar to those of the host (Wei et al., 2020, 2023). However, Interestingly, similar to reporting results forward (Wei et al., 2020, 2023), the differential gut microbiome of the hybrid offspring displayed clear advantages over those of the parents. The hybrid offspring not only inherited the dominant gut microbiome of the parents, such as *g_Prevotellaceae UCG-003, f_Bacteroidales RF16 group, Ambiguous_taxa, g_Alloprevotella* from the paternal parents, *g_Alistipes* from the maternal parents. However, there were also many significant advantages in the hybrids in comparison to the parents, such as *g_Prevotellaceae UCG-003*, *f_Bacteroidales RF16 groupg_uncultured bacterium*, *g_Roseburia*, *g_Succinivibrio*, *g_Prevotellaceae UCG-001*, *g_Prevotella 1*, and *g_Lachnospiraceae UCG-006*. Some research revealed that members of *Prevotellaceae* and *Succinivibrio* can degrade plant cell wall polysaccharides (Dai et al., 2015; Gatya et al., 2022) and proteins (Cowieson and Bedford, 2009; Brinkman et al., 2011). *Prevotella*, acting as a catalyst for proteins (Xue et al., 2020), influences the metabolism of the serum amino acids produced by the host (Xue et al., 2020). Additionally, both *Prevotellaceae* and *Succinivibrio* are renowned producers of succinic acid, which can improve glucose homeostasis by activating intestinal gluconeogenesis (De Vadder et al., 2016). *Prevotellaceae* can also produce propionate through the succinate or acrylate pathways (Purushe et al., 2010). Moreover, *Succinivibrio* can produce acetic acid, propionic acid, and lactic acid, which influence the energy metabolism of the host (De Menezes et al., 2011; Bryant, 2015; Liu et al., 2017). The results indicated that there was a positive correlation between the abundances of *Prevotellaceae UCG-003*, *Prevotellaceae UCG-001*, *Succinivibrionaceae UCG-002*, and *Succinivibrio*, and the rate of feed collection of the animals (Auffret et al., 2020; Zhang et al., 2021; Qiu et al., 2022). *Roseburia*, *Lachnospiracea*, and *Bacteroides* have the ability to butyric acid through the fermentation of various plant polysaccharides, thereby regulating metabolism, immune responses, and the growth of colon cells (Meehan and Beiko, 2014; Ling et al., 2020; Pereira et al., 2021). In addition to butyric acid production, *Lachnospiraceae* can also produce β-glucuronidase (Gloux et al., 2011). Research has shown that *Roseburia* may play a significant role in weight gain (Kyriachenko et al., 2019). The *Bacteroidales RF16 group* is widely present in yaks, beef cattle, and dairy cows (Dan et al., 2016; Popova et al., 2017; Schären et al., 2018), and it potentially plays a role in regulating the feed efficiency in Angus breeds (Fonseca et al., 2023). Transitioning from an animal-based diet to a plant-based diet has been associated with an increase in the relative abundance of *Alistipes* in the *Rikenellaceae* family (David et al., 2014), and it may play a role in degrading the polysaccharides derived from plants (Peng et al., 2015). In conclusion, these microbial communities may serve as key contributors to the advantages of hybrid hosts and may produce short chain fatty acids in the body to promote host health, energy metabolism, and the rate of feed collection, thereby enhancing the growth of deer pilose antlers.

The growth of deer pilose antlers is influenced by various factors, such as genetic factors, dietary conditions, and hormones (Fennessy and Suttie, 1985; Anderson et al., 2019). Additionally, pilose antler growth is regulated by various cell growth factors, such as the “Wnt signaling pathway,” “PI3K-Akt signaling pathway,” “MAPK signaling pathway,” and “TGF-β signaling pathway,” all of which have been confirmed to be associated with the deer pilose antler growth (Zhang et al., 2023). Interestingly, a metagenomic analysis revealed that the “Wnt signaling pathway,” “PI3K-Akt signaling pathway,” and “MAPK signaling pathway – yeast” were significantly enriched in hybrid deer, which suggested that these pathways may play a crucial role in the production of pilose antlers in hybrid deer. Furthermore, the levels of testosterone exert an influence on the growth of deer pilose antler tissues (Sadighi et al., 2001), which can potentially be mediated by local aromatization to estradiol (de Bournonville et al., 2020). Studies on the fecal testosterone levels in male water deer (*Hydropotes inermis*) have also revealed a specific relationship between the content of fecal testosterone and the growth of pilose antlers (Weerasekera et al., 2020). compared with those of the parental deer, the fecal samples of the hybrid deer were enriched in various steroid hormones, such as 7α-hydroxytestosterone, estriol, and estradiol, and were significantly enriched in the “Steroid hormone biosynthesis metabolic pathway.” This may be related to the high level of pilose antler production in hybrid deer. In addition, there was a significant enrichment of the metabolic product arachidonic acid in hybrid deer. Studies have reported that the addition of exogenous arachidonic acid to purified rat Leydig cells can promote the testosterone production (Romanelli et al., 1995), which may indirectly regulate the growth of red deer pilose antlers. Moreover, arachidonic acid plays a significant role in the regulation of animal reproduction (Marei et al., 2014).

Compared with those of the parents, the fecal samples of hybrid red deer contained significantly more metabolites. In addition to steroid hormones, these metabolites are primarily related to the metabolism of B vitamins and some amino acids, such as tryptophan, valine, leucine, isoleucine, and lysine. Metagenomic analysis also revealed that these metabolic pathways were significantly enriched in the hybrid red deer. Vitamin B families play important roles in promoting animal growth and development, maintaining normal physiological functions, and regulating immune and inflammatory responses (Mooney et al., 2009; Lanska, 2012; Thakur et al., 2017). Castagnino et al. (2018) reported that the apparent ruminal biosynthesis of riboflavin was positively correlated with the degradation of neutral degrant fibers, duodenal microbial N flow, and the molar ratio of the acetic acid concentration in the rumen, and negatively correlated with the rumen pH. Thus, it was deduced that riboflavin was primarily synthesized by the bacteria that degrade fiber. Interestingly, a similar phenomenon was identified in this study, where a variety of bacteria capable of degrading cellulose were also positively correlated with riboflavin. In addition, deficiencies in riboflavin and vitamins B6 and B5 will lead to decreases in animal weight, the rate of feed collection and litter size (Frank et al., 1984; Jang Hua, 2004; Mayengbam et al., 2020). Branched chain amino acids, such as valine, leucine and isoleucine, not only maintain energy homeostasis by providing nitrogen substrates and a carbon framework, but also regulate the biosynthesis of glucose, lipids and proteins; lactation in female animals; and hormone and lipid metabolism by providing signaling molecules (Kim et al., 2022). Tryptophan and its metabolites have a variety of physiological functions, such as increasing the rate of feed collection, neuromodulation, immune regulation and intestinal homeostasis (Yao et al., 2011; Marsland, 2016; Zhang Wenjie et al., 2020). Moreover, lysine is widely used in ruminant production due to its ability to promote their growth and development, improve milk production and quality, reduce feed costs, promote digestion and absorption, and enhance immunity (Hosford et al., 2015; Giallongo et al., 2016). Lysine can also fermentation pattern in the rumen, increase the concentration of ruminal volatile fatty acids (VFA), and change the proportion of levulinic acid, thereby indirectly stimulating the growth of microorganisms (Zhang and Xu, 2013).

The interaction between the gut microbiome and metabolites in hybrid deer was explored in more detail via network analysis, revealing a significant positive correlation between the metabolites related to “Steroid biosynthesis,” “Tryptophan metabolism,” “Valine, leucine and isoleucine biosynthesis,” “Lysine degradation,” and “Arachidonic acid metabolism” and the abundances of the *Bacteroidales RF16 group*, *Succinivibrio*, *Prevotella1*, and *Prevotellaceae UCG-003*. Additionally, metabolites associated with “Riboflavin metabolism,” “Vitamin B6 metabolism,” and “Pantothenate and CoA biosynthesis” were primarily positively correlated with the *Bacteroidales RF16 group* and *Prevotellaceae UCG-003*. Previous studies have proven that these gut microbiome can degrade plant cell walls and produce short chain fatty acids, exerting important regulatory effects on the growth and development of animal bodies and immunity (Flint et al., 2012; Bryant, 2015; Dai et al., 2015; De Vadder et al., 2016; Qiu et al., 2022). Therefore, we hypothesized that the *Bacteroidales RF16 group*, *Prevotellaceae*, and *Succinivibrio* may promote the biosynthesis of steroids, amino acids, vitamin B, and other metabolites through the production of short chain fatty acids, thus providing favorable conditions for the growth and development of hybrid red deer. Overall, this study revealed the complex interaction network between bacteria and metabolites in hybrid red deer, which provides in-depth insights for further understanding the impact of hybridization on animal physiology and ecology. However, further studies on animals are needed to confirm these findings, which have highly significant implications for the development of pilose antler production and aquaculture, as well as for providing a broader understanding of the relationship between the animal microbiome and host health.

## 5 Conclusion

In this study, the gut microbiome, as well as the function and metabolites of hybrid red deer offspring, exhibit significant advantages over those of their parents, which suggests that hybridization of the host may have the same remodeling effect on the gut microbiome of red deer. Furthermore, the *Bacteroidales RF16 group*, *Prevotellaceae* and *Succinivibrio*, which are significantly enriched in hybrid red deer, may promote the biosynthesis of metabolites related to “Steroid biosynthesis,” “Tryptophan metabolism,” “Valine, leucine and isoleucine biosynthesis,” and “Riboflavin metabolism,” “Vitamin B6 metabolism.” These metabolites may be the key factors leading to its high-yielding pilose antlers and good growth, as well as strong levels of immunity. The results of this study add to the hybridization data on the red deer gut microbiome and provide reference data for further screening potential probiotics and performing microbial-assisted breeding to promote the pilose antler growth and body health.

## Data availability statement

The datasets presented in this study can be found in online repositories. The names of the repository/repositories and accession number(s) can be found at: https://www.ncbi.nlm.nih.gov/, PRJNA1065901. https://www.ncbi.nlm.nih.gov/, PRJNA1066509.

## Ethics statement

The animal study was approved by Institutional Animal Care and Use Committee of the Chongqing Medical and Pharmaceutical College, Chongqing, China (KCB-2220031). The study was conducted in accordance with the local legislation and institutional requirements.

## Author contributions

LW: Conceptualization, Formal analysis, Methodology, Writing – review & editing. BZ: Software, Visualization, Writing – original draft. BL: Data curation, Resources, Writing – original draft. WG: Data curation, Resources, Writing – original draft. ZM: Validation, Writing – original draft. YG: Investigation, Writing – original draft. YL: Funding acquisition, Project administration, Supervision, Writing – review & editing.

## Footnotes

^1^ https://www.microbiomeanalyst.ca/
