# Supplementary figures and images for: Hybridization alters red deer gut microbiome and metabolites
Source: Front Microbiol. 2024 May 3;15:1387957. doi: 10.3389/fmicb.2024.1387957 (PMC11112572; doi:10.3389/fmicb.2024.1387957)

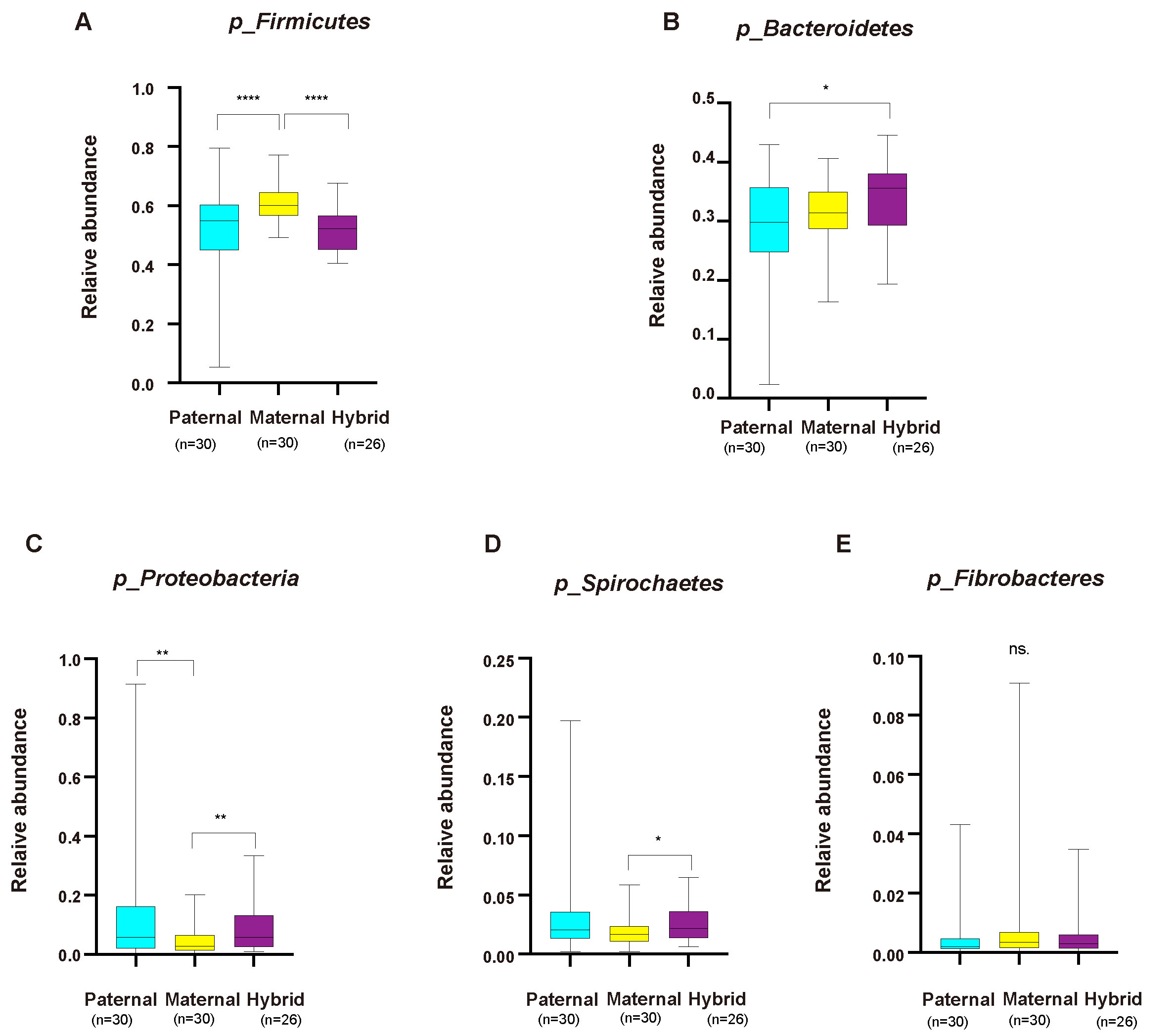

Supplement: Supplementary file 6 [file Image_1.jpg]

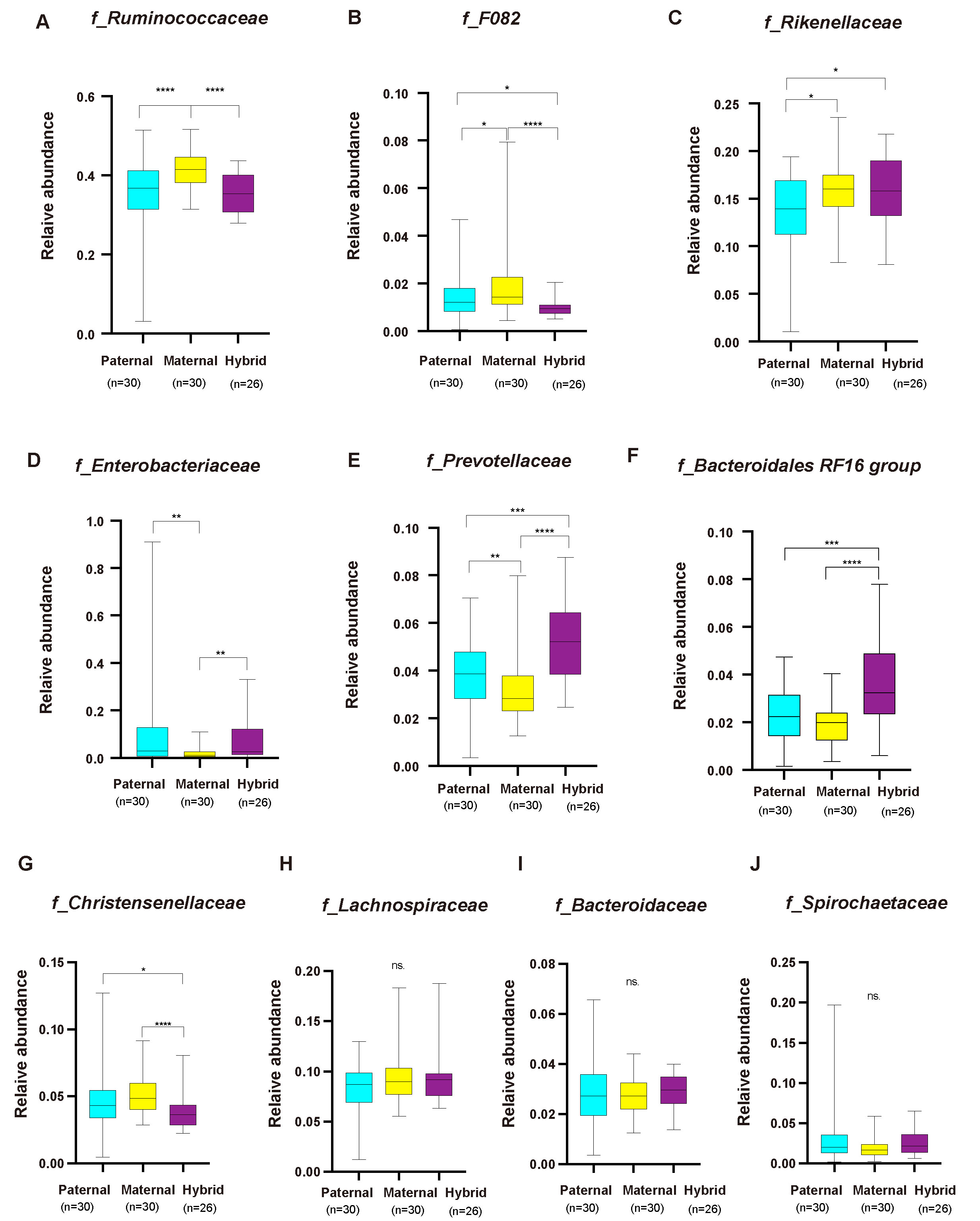

Supplement: Supplementary file 7 [file Image_2.jpg]

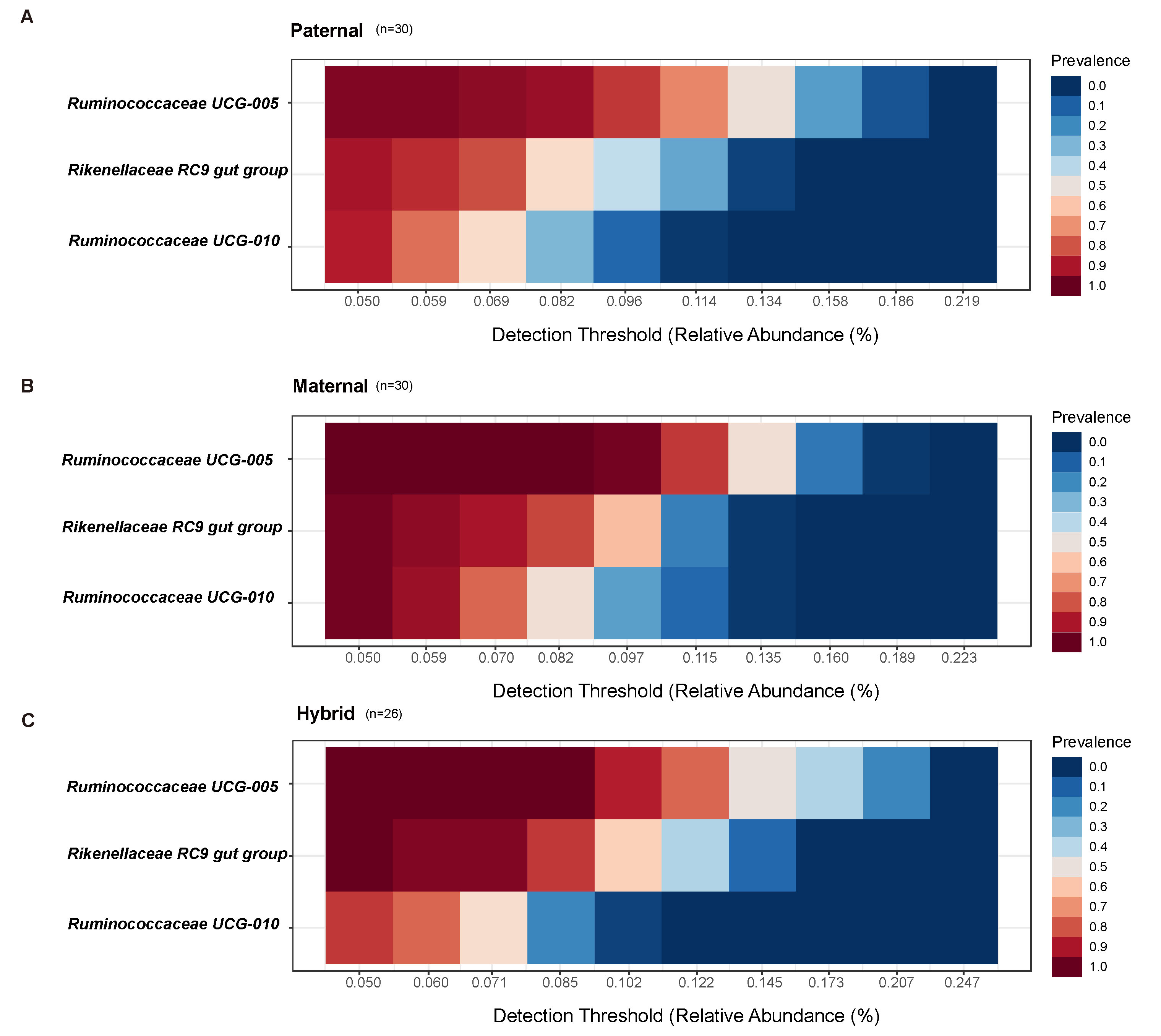

Supplement: Supplementary file 8 [file Image_3.jpg]

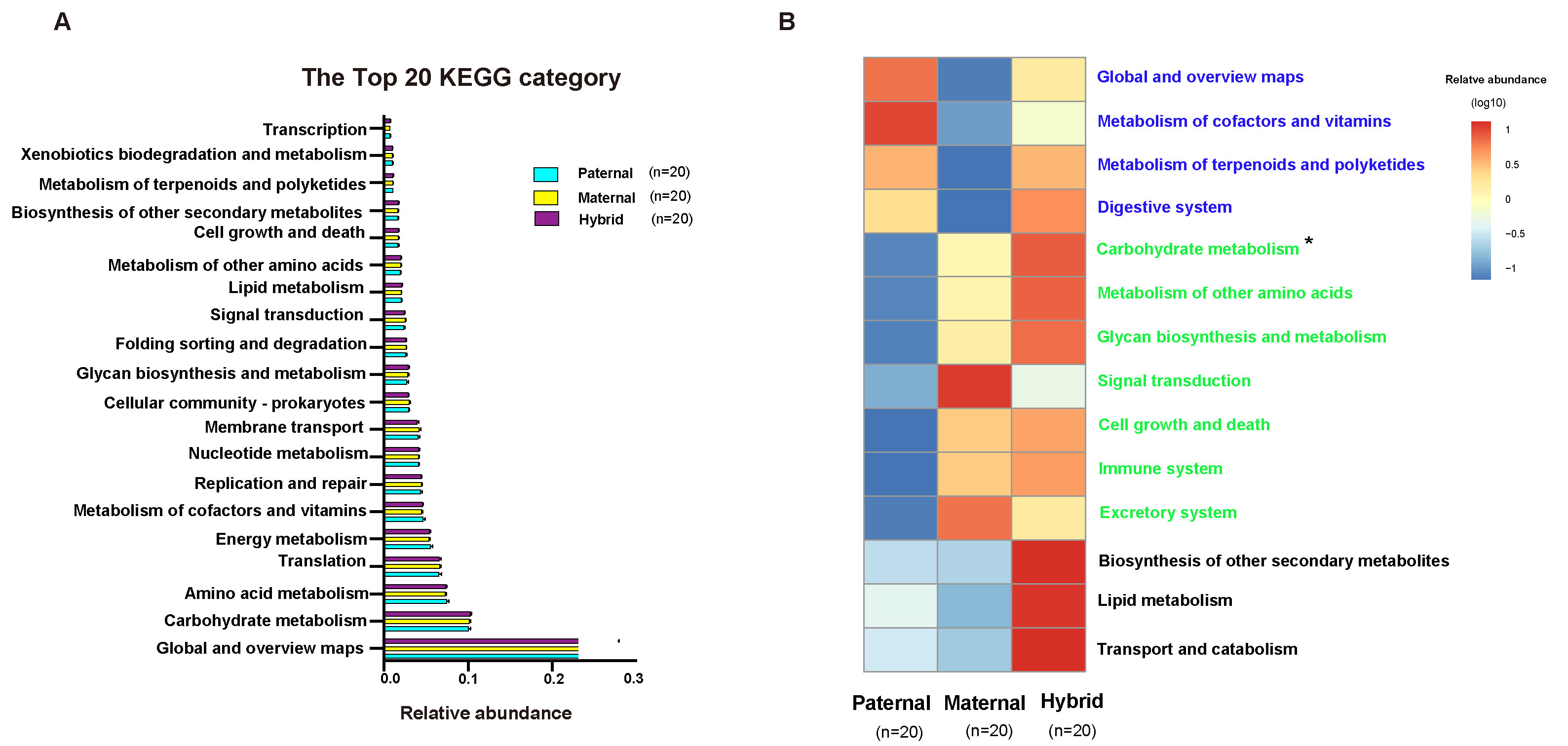

Supplement: Supplementary file 9 [file Image_4.jpg]

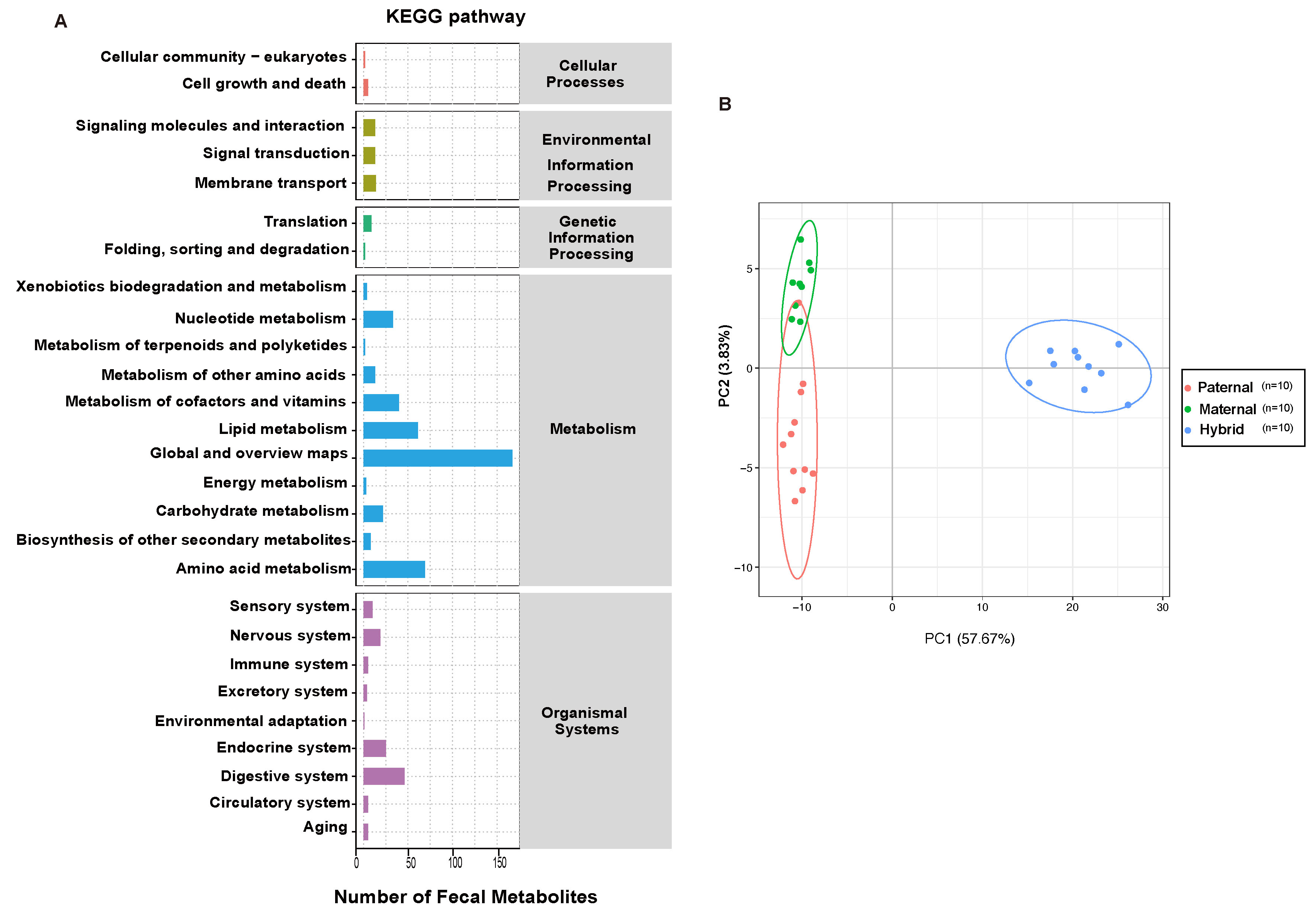

Supplement: Supplementary file 10 [file Image_5.jpg]
